# Genetic Diversity and Population Structure Analysis of Seven Duck Populations of Bangladesh Using Microsatellite Markers

**DOI:** 10.3390/vetsci13040407

**Published:** 2026-04-21

**Authors:** Pranto Saha, Krishna Chandra Barman, Minjun Kim, Dongwon Seo, Md. Munir Hossain, Seung Hwan Lee, Md Azizul Haque, Mohammad Shamsul Alam Bhuiyan

**Affiliations:** 1 Department of Animal Breeding and Genetics, Bangladesh Agricultural University, Mymensingh 2202, Bangladesh; pranto.1803140@bau.edu.bd (P.S.); krishnabarmon38@gmail.com (K.C.B.); mmhossain@bau.edu.bd (M.M.H.); 2 Division of Animal and Dairy Science, Chungnam National University, Daejeon 34134, Republic of Korea; mjkim6023@naver.com (M.K.); slee46@cnu.ac.kr (S.H.L.); 3 TNT Research Co., Ltd., Sejong City 30141, Republic of Korea; dwseo@tntresearch.co.kr; 4 Department of Biotechnology, Yeungnam University, Gyeongsan 38541, Republic of Korea

**Keywords:** molecular marker, genetic diversity, population structure, phylogeny, duck, Bangladesh

## Abstract

The objectives of this paper were to assess the genetic diversity, population structure, genetic differentiation, and phylogenetic relationships among seven duck populations using 14 microsatellite (MS) markers. This paper included 176 individuals representing seven duck populations of Bangladesh: indigenous duck (BLD), Nageswari (NAG), Rupali (RUP), Jinding (JIN), Pekin (PEK), BAU Black and White (BWC), and BAU White (WHC). A total of 133 alleles were observed with a mean of 9.50 alleles per locus. Genetic diversity was evaluated using measures such as allele frequency, observed and expected heterozygosity, and Shannon’s information index with average values of 5.44 ± 0.31, 0.59 ± 0.02, 0.64 ± 0.02, and 1.28 ± 0.05, respectively. Population differentiation and inbreeding analysis (F-statistics) indicated moderate genetic diversity and a slight degree of inbreeding across populations. Analysis of molecular variance indicated that 75% of the total genetic diversity was attributable to the within-population variation, whereas 9% and 16% were attributed to the variation among individuals and population differentiation, respectively. Indigenous duck populations (BLD, NAG, and RUP) had a close genetic relationship with JIN ducks and an intermediate relationship with two crossbreds (BWC and WHC), and the highest genetic distance was observed with PEK ducks. Neighbor-joining phylogenetic analysis revealed that the Bangladeshi indigenous duck populations formed a single cluster, while the two crossbreds (BWC and WHC) and PEK exhibited their distinct genetic identities in separate clusters. Furthermore, structure analysis at K = 2 to 5 confirmed the distinct genetic architecture (ΔK = 4.00) of the studied duck populations. This paper provides important insights into genetic diversity measures and population differentiation that will be helpful in future genetic improvement, conservation initiatives, and the design of appropriate breeding programs.

## Simple Summary

Bangladesh has several indigenous and improved duck populations that play an important role in rural livelihoods, food security, and poultry production. However, information about their genetic diversity and relationships is still limited, which makes it difficult to design effective conservation and breeding programs. In this paper, we analyzed the genetic diversity and population structure of seven duck populations of Bangladesh using microsatellite markers. A total of 176 ducks representing indigenous ducks (Non-descript Deshi, Nageswari, and Rupali), exotic breeds (Jinding and Pekin), and two crossbred duck populations (BAU Black and White and BAU White) were evaluated. The results showed moderate genetic diversity among the duck populations with most of the genetic variation occurring within populations rather than between them. These findings can support future conservation efforts, genetic improvement strategies, and the development of sustainable duck-breeding programs in the country.

## 1. Introduction

Ducks are an important poultry genetic resource of Bangladesh, playing a significant role in the rural economy, food security, livelihoods, and women’s employment. Small-scale duck farming is a common practice in the rural areas of Bangladesh with a few exceptions in the low-lying marshy and coastal regions where bigger flocks are being maintained [1]. The total duck population in Bangladesh is about 68.26 million [2], providing up to 22–25% of total poultry egg production [3]. Prolonged productive life, better foraging capability, adaptability to adverse environmental conditions, and less prone to disease are the key reasons behind duck farming [1,4]. The duck genetic resource of Bangladesh comprises mostly non-descript indigenous, crossbreds, or upgrades, and some egg-type exotic breeds like Khaki Campbell, Jinding, and Indian Runner. In the recent past, Pekin has been introduced as a meat-type duck even though their production performances and adaptability under the traditional semi-intensive husbandry practices is not satisfactory [5].

Crossbreeding or upgrading programs have been used globally in order to increase heterosis or hybrid vigor in ducks along with better adaptability under certain environments [4,6,7]. The BAU Black and White crossbred duck has been developed recently through reciprocal crossing between Pekin and Nageswari breeds up to seven generations to increase the production of meat without compromising egg production [8]. The gene allele frequency has already been fixed for these populations and is already established as a popular variety of Bangladesh among the farmers. Although the phenotypic features of F_1_ crossbreds were similar to those of parental Nageswari ducks due to the influence of the extended E locus [9], however, the plumage color varied in subsequent generations. The Bangladesh Livestock Research Institute also developed a white colored indigenous duck variety (Rupali) through selective breeding [10]. Therefore, it is hard to differentiate morphologically similar duck populations of Bangladesh based on phenotypes, which is crucially important for breed discrimination and traceability. A molecular marker-based study could give a relevant answer for disclosing the genetic architecture and differentiating the available duck genetic resources of Bangladesh.

Molecular genetic characterization provides insightful information on population diversity, genetic structure, evolutionary, and ecological investigations, which are prerequisites for breed discrimination or establishing a new breed or variety with uniqueness [11]. Microsatellites (MSs) are highly polymorphic and potent molecular markers that are extensively applied for the genetic characterization of diverse livestock and poultry populations, including duck [12,13]. Hence, a panel of recommended MS markers has been routinely and successfully utilized for analyzing the molecular variability of different duck populations [14,15,16]. However, to the best of our knowledge, there has been limited genetic information available about Bangladeshi ducks, and no comprehensive study has yet been conducted to assess the genetic diversity and population structures of all duck populations available in Bangladesh. Therefore, the objectives of this paper were to assess the intra- and inter-population genetic variability, genetic relatedness, and population structure of seven duck populations of Bangladesh using 14 selected microsatellite markers.

## 2. Materials and Methods

### 2.1. Sampling and DNA Extraction

In total, 185 unrelated blood samples were collected from seven duck populations of Bangladesh from Mymensingh, the Bangladesh Livestock Research Institute (BLRI), and the Bangladesh Agricultural University research farm. Approximately 2–3 mL of blood was taken aseptically from the wing vein using a venoject tube. The genomic DNA extraction was performed using the AddPrep Genomic DNA extraction kit (Add Bio Inc., Daejeon, Republic of Korea). The NanoDrop spectrophotometer (Model ND2000, Thermo Fisher Scientific, Wilmington, DE, USA) was used to quantify and purity assessment of the extracted DNA. Finally, 176 samples (BLD = 12, NAG = 32, PEK = 32, JIN = 14, RUP = 18, BWC = 36, and WHC = 32) were selected for further study based on the extracted DNA’s concentration and purity.

### 2.2. DNA Amplification and Microsatellite Genotyping

#### 2.2.1. Selection of Primer and Polymerase Chain Reaction Amplification

Fourteen highly polymorphic MS markers were selected from the earlier studies of Seo et al. [14] and Sultana et al. [17]. These markers are distributed across the genome on seven different chromosomes, where multiple markers on each chromosome are well spaced and genetically independent [14]. Microsatellite loci were amplified using multiplex polymerase chain reaction (PCR) in a 20 µL reaction volume using four distinct fluorescent dye-labeled forward primers (Table 1). The components of the PCR master mix were 2.0 μL gDNA (~50 ng DNA), 10 μL AddStart Taq Master (2x conc.) multiplex PCR buffer (Add Bio Inc., Daejeon, Republic of Korea), and 0.5 μL of 10 pmol each forward and reverse primer, and the required volume of deionized water to adjust the final volume. Amplification was carried out in a C1000 Thermal Cycler (Bio-Rad, Irvine, CA, USA) with an initialization at 95 °C for 10 min, which was followed by 35 cycles of denaturation at 95 °C for 30 s, annealing at 60 °C for 30 s, extension at 72 °C for 30 s, and a final extension at 72 °C for 10 min. PCR products were electrophoresed in 2.0% agarose gel, and images were captured using a Bio-Rad gel documentation system (Bio-Rad, Irvine, CA, USA).

#### 2.2.2. Microsatellite Genotyping

The genotyping reaction was performed in a 10 μL total volume containing 1.0 μL of diluted PCR product and 9 μL of Hi-Di formamide (Applied Biosystems, Foster City, CA, USA) that includes 0.1 μL of the GeneScan-500LIZ size standard (Applied Biosystems, USA). The reaction volume was denatured at 95 °C for 5 min. Fragment analysis was performed by capillary electrophoresis array using the Genetic Analyzer 3130 xl (Applied Biosystems, Foster City, CA, USA). The allele sizes were determined by GeneMapper software ver. 4.1 (Applied Biosystem, USA), and accordingly, the allele score was recorded.

### 2.3. Data Analysis

The genetic diversity measures, such as the average number of alleles (Na), the effective number of alleles (Ne), the observed heterozygosity (Ho), and the expected heterozygosity (He) for each locus across populations and for each population including all loci were computed using GenAlEx ver.6.5 [18]. Additionally, Shannon’s information index (I), the fixation index (F), and Wright’s [19] fixation indices F_IS_, F_IT_, and F_ST_ for each marker across populations were computed using the variance-based method of Weir and Cockerham [20] with the aforementioned software. Arlequin V3.5 software [21] was employed to assess pairwise genetic differentiation using Wright’s F statistics and to conduct an analysis of molecular variance (AMOVA). The pairwise F_ST_ and Nei genetic distances between seven duck populations were calculated using the StAMPP package(version 1.6.3) in R [22]. In order to infer the evolutionary relationships among seven duck populations, an unrooted neighbor-joining phylogenetic tree was constructed according to the standard genetic distance of Nei with 1000 bootstraps using POPTREE2 software [23]. Furthermore, the STRUCTURE analysis [24] was implemented for inferring the population structure from multi-locus genotypes utilizing a model-based clustering method. This method uses Markov chain Monte Carlo (MCMC) simulations of 100,000 iterations with an initial burn-in period of 20,000 generations. A number of hypothetical population clusters (K) ranging from 2 to 5 was employed where the optimum number of K (ΔK = 4.00) was calculated from the lowest cross-validation (CV) error.

## 3. Results

### 3.1. Polymorphism of Microsatellite Markers

A total of 133 alleles were detected in the 14 microsatellite markers with an average of 9.50 alleles per locus (Table 2). The number of alleles differed largely among the 14 markers, where the CAUD040 marker possessed the highest alleles (*n* = 26) and the AMU3 had the lowest alleles (*n* = 4). Considering all MS loci, the expected (He) and observed heterozygosity (Ho) averaged 0.64 and 0.58, respectively. The lowest He (0.52) was found in the CAUD111 locus, and the CAUD040 had the highest He estimate (0.88). All 14 MS loci were in HWE agreement (*p* > 0.05) except APH04, AMU3, and CAUD069 loci. The average within-population inbreeding coefficient (F_IS_) was 0.09 where seven loci (CAUD111, APH04, APH08, CAUD005, CAUD086, CAUD035, and CAUD048) had negative F_IS_ values, and the remaining seven markers possessed some degree of inbreeding at the population level. The average total population inbreeding coefficient (F_IT_) was 0.22, and the average genetic distance among populations was 0.16.

### 3.2. Genetic Diversity Across Seven Duck Populations

Genetic diversity measures of seven different duck populations of Bangladesh are shown in Table 3. Considering all duck populations, the average number of alleles (Na), average number of effective alleles (Ne), and Shannon information index (I) were 5.44 ± 0.31, 3.46 ± 0.19, and 1.28 ± 0.05, respectively. The NAG duck population had the highest Na (6.29 ± 1.08), Ne (4.14 ± 0.65), and I (1.46 ± 0.12) values, and the corresponding measures were found to be the lowest in PEK ducks (4.71 ± 0.77, 2.49 ± 0.41, and 0.98 ± 0.13, respectively). The average observed heterozygosity (Ho) and expected heterozygosity (He) were 0.59 ± 0.02 and 0.64 ± 0.02 with a range of 0.51 ± 0.06 to 0.64 ± 0.06 and 0.51 ± 0.05 to 0.71 ± 0.03, respectively. Although every population had lower observed heterozygosity than expected, none was below 0.50. Overall, the average fixation index (F) for all populations was 0.09 ± 0.03, ranging between 0.01 ± 0.07 (PEK) and 0.15 ± 0.09 (BLD and NAG).

### 3.3. Genetic Distance and Relationship

**Figure 1:**
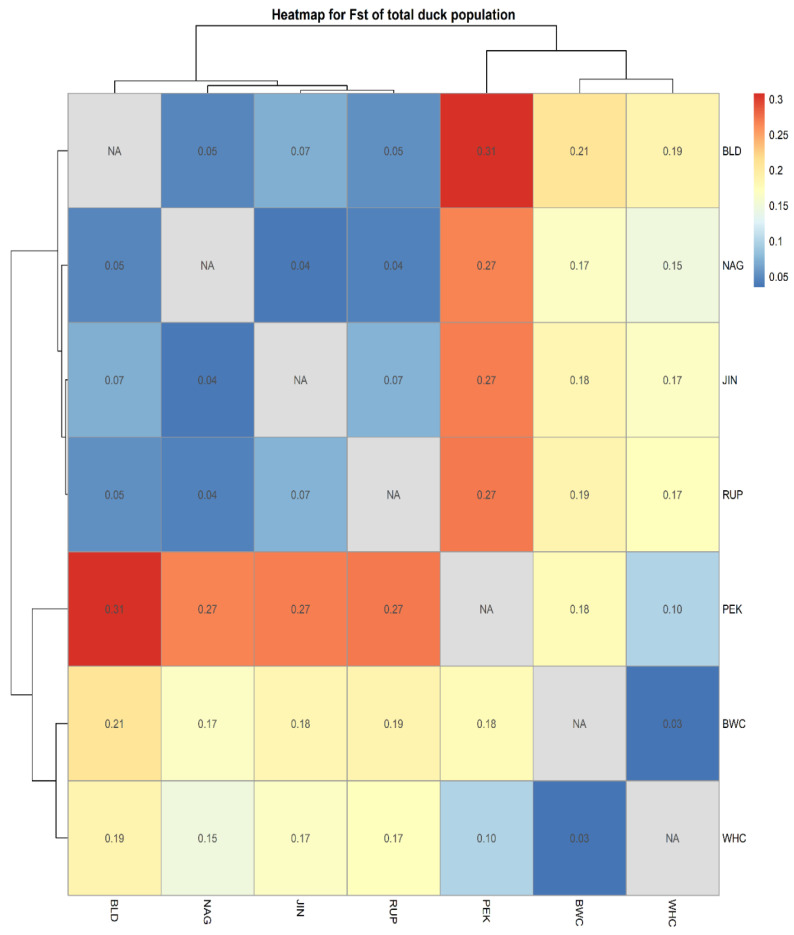
Heatmap represents the pairwise F_ST_ (above diagonal) and Nei genetic distance (below diagonal) values among seven duck populations including Indigenous and Jinding (light blue to deep blue), Pekin (red), and crossbred duck (accent gold) of Bangladesh.

Table 4 illustrates the pairwise genetic distance (F_ST_) among the studied populations. The genetic distance between populations ranged from 0.035 to 0.308. The highest genetic distance was displayed between BLD and PEK (0.308), while the lowest distance was observed between BWC and WHC (0.035). Bangladeshi duck populations (BLD, NAG, and RUP) had a close genetic relationship with egg type JIN duck (0.046 to 0.071), while they had a moderate relationship with crossbred BWC and WHC ducks (0.147 to 0.208), and the largest distance was observed with PEK ducks (0.308). Furthermore, the analysis of molecular variance (AMOVA) indicated that 16% of the genetic variation was attributed to differences among seven duck populations, with 9.0% of variation occurring among individuals, while the remaining 75% of variation was found within populations (Table 5). The heatmap algorithm that was extracted from the genetic relationship matrix’s hierarchical clustering also demonstrated similar results to both pairwise distance (above diagonal) and Nei’s genetic distance (below diagonal). In both cases, PEK was the most genetically distant group, and Bangladeshi BLD, RUP, and NAG ducks were found to be the most related (Figure 1).

### 3.4. Phylogenetic Relationships and Structure Analysis

**Figure 2:**
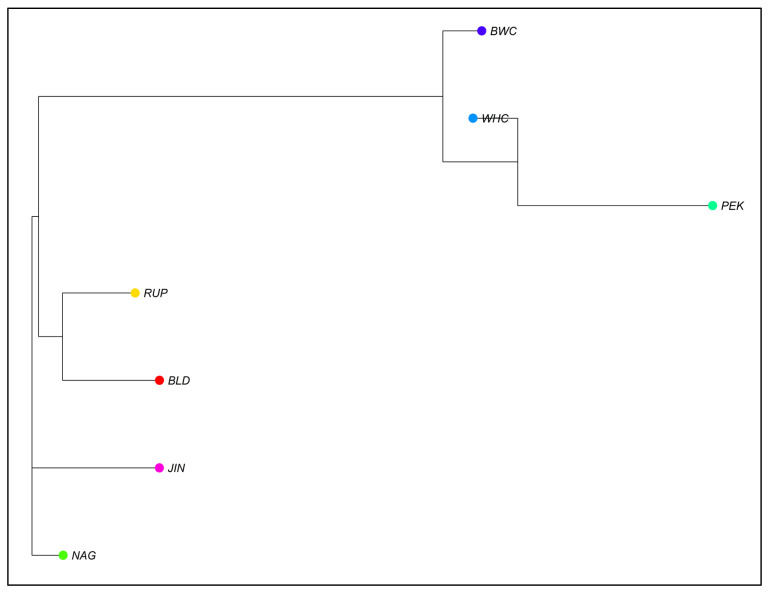
Neighbor-joining (NJ) phylogenetic tree constructed using genetic distance per population (Nei, 1972) illustrating relationships among seven different duck populations of Bangladesh. BLD, Non-descript Deshi; RUP, Rupali; NAG, Nageswari; PEK, Pekin; WHC, BAU White Crossbred; BWC, BAU Black and White Crossbred; and JIN, Jinding.

**Figure 3:**
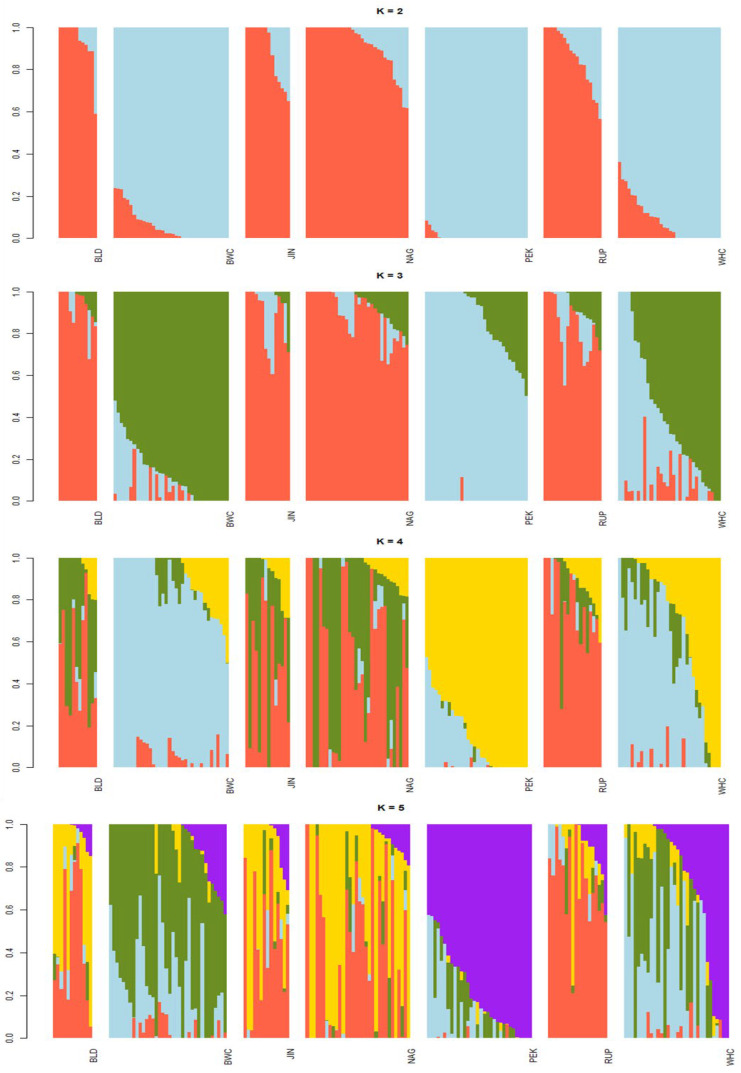
Population structure analysis in different duck populations of Bangladesh for K values ranging from 2 to 5. BLD, Non-descript Deshi; RUP, Rupali; NAG, Nageswari; PEK, Pekin; WHC, BAU White Crossbred; BWC, BAU Black and White Crossbred; and JIN, Jinding.

The phylogenetic tree based on Nei’s distance matrix is shown in Figure 2, where RUP, BLD, JIN, and NAG made a single cluster indicating a common origin with minimum genetic distance. PEK was positioned distinctly in the tree from the other six duck populations. On the other hand, the BWC and WHC populations are grouped into a separate cluster and maintained a distance from both clusters (Figure 2). The genetic structure of seven duck populations were investigated using STRUCTURE software with K values ranging from 2 to 5 (Figure 3). At K = 2, Bangladeshi three duck populations (BLD, RUP, and NAG) along with JIN formed the first group, whereas BWC, WHC, and PEK made the second group. The optimum K value (K = 4) was determined based on the outputs of the lowest cross-validation error. The output on K = 4 seems most reliable (Figure 3), and every duck population was clearly distinguished from the others. Both WHC and BWC showed distinct genetic characteristics from PEK and other indigenous duck populations, along with JIN, though containing some traces of genetic admixture from other populations.

## 4. Discussion

In case of genetic analysis, both microsatellite markers and SNP chips are powerful tools but differ in their applications and advantages. Microsatellite markers are highly used in genetic diversity studies due to their highly polymorphic nature, offering a cost-effective approach, and they are particularly valuable for investigating population structure and genetic diversity [11,12,13]. On the other hand, SNP chips offer a high-throughput, genome-wide approach. These chips are advantageous for large population studies due to their ability to assess a wide range of genetic variants across the genome. However, HD-SNP analysis is limited in duck studies [14].

Ducks are among the many poultry species for which microsatellite markers (MSs) have been used extensively to study genetic diversity, population structure analysis, develop traceability systems, and design breeding programs for effective conservation and utilization [25]. In this paper, the genetic characterization of the predominantly available seven duck populations of Bangladesh was investigated using 14 selected highly polymorphic markers. Although BLD and JIN were represented by relatively fewer individuals, this reflects practical constraints in the Bangladesh context rather than a study design limitation. These populations are less accessible and are often maintained in small, scattered systems, which makes large-scale sampling difficult. In addition, limited research resources, logistical challenges in covering diverse geographic regions, and the absence of well-defined population records further restricted sample collection. However, our findings are supported by the findings of Yacouba et al. [26], Veeramani et al. [27], and Wolc et al. [28]. Our results are comparable to those of Veeramani et al. [27] who detected a total of 222 alleles using 23 MS loci with an average of 9.65 ± 0.95 alleles per locus in six Indian duck populations. Carcò et al. [29] identified a total of 261 alleles, averaging 11.36 alleles per locus among two Italian and two Polish duck breeds, which exceeds the findings of this paper. Hariyono et al. [15] identified 153 alleles across 22 MS loci in the Indonesian local duck population, yielding an average of 6.96 alleles per locus, which is lower than our results.

The number of alleles per locus, observed and expected heterozygosity, polymorphic information content (PIC), and fixation index are important parameters to quantify the genetic diversity within and between populations [14]. Similar to this paper, the average effective number of alleles (Ne) reported by Debnath et al. [16] in the indigenous duck of Tripura state in India was 3.54 ± 0.53. However, higher Ne values were found in two different populations, 5.37 and 4.00, respectively [13,30]. Additionally, the Indian duck population had 1.95 effective alleles per locus, which was lower than our findings [31]. On the other hand, a lower average Ne was observed from our results [12,15,29] due to a different panel of MS markers and the population studied.

The observed heterozygosity (Ho) in our result indicates a good balance of genetic diversity except for the CAUD069 marker. In terms of Ho, Wolc et al. [28] reported similar results in six Polish duck populations ranging between 0.515 and 0.589. The average Ho (0.546) of this paper was aligned with the findings of Das et al. [31] in indigenous duck populations of northeastern India. Moreover, Indonesian local duck populations (0.465) and South and East Asian ducks (0.492) resulted in comparatively lower Ho values [14,32]. The observed heterozygosity in the Indian crossbred duck population was 0.07 ± 0.04, which is remarkably low compared to this paper [33]. On the other hand, Lai et al. [13] reported higher results in Tsaiya ducks. It is difficult to compare directly among the relevant studies due to differences in genetic background and the use of different genetic markers [34]. The differences between the previous studies and current findings might be due to differences in the sample size, population architecture, number of MS markers used, population-specific alleles, and/or allele scoring bias (null allele or allele drop out) [35]. Shannon’s information index, a good measure of the biodiversity of a population, was 1.153 [12], 1.184 ± 0.112 [16], and 0.926 ± 0.06 [33] in Chinese native ducks, Indian crossbred and Indian indigenous duck populations, respectively, and these findings are aligned with those of this paper.

The distribution of genetic variation within and among populations is measured by three F-statistics: F_IS_, F_IT,_ and F_ST_. Lai et al. [13] reported that the overall within population inbreeding (F_IS_), total inbreeding (F_IT_), and mean genetic distance (F_ST_) in eight Chinese duck populations were 0.043 (−0.072 to 0.304), 0.179 (0.105 to 0.298), and 0.233 (0.095 to 0.397), respectively, which is comparable to the findings in this paper. A panel of 22 microsatellite markers utilized by Maharani et al. [32] included 13 markers similar to those in this paper. They reported F_IS_, F_IT_, and F_ST_ values of 0.112, 0.197, and 0.093, respectively, in Indonesian duck populations, which are consistent with the findings in this paper, except for the F_ST_ value. Further, Veeramani et al. [27] reported that the overall mean F_IS_, F_IT,_ and F_ST_ values were 0.137, 0.339, and 0.234, respectively, in six Indian duck populations, which is higher than those reported in this paper. Our results showed low to moderate F-statistic values across the loci, indicating that a moderate genetic differentiation existed among the studied populations.

Large (>0.15) or moderate (0.05 to 0.15) genetic variation between the investigated populations is suggested by the evaluation of pairwise genetic distance (F_ST_) [19]. In this paper, the lowest F_ST_ was found between BWC and WHC because both populations were crossbreds and their parental lines were Pekin and Nageswari ducks. These two crossbred populations (BWC and WHC) maintain a larger genetic distance with both the indigenous population (20.8%) and Pekin (18.6%). In addition, a lower genetic distance among BLD, NAG, and RUP implies these genotypes belong to the same origin. Similar to our results, Hariyono et al. [15] stated genetic distance ranging from 5.4% to 29.9% in Indonesian local duck populations. Wu et al. [36] reported Nei’s standard genetic distance varied from 0.075 to 0.143 among 11 Beijing duck lines and 2 Cherry Valley duck lines using 18 microsatellite markers, which is also within the range of this paper. In addition, the current results of AMOVA based on the allelic distance matrix are supported by the study of Lai et al. [13] who reported variation within population, among populations, and among individuals as 76%, 19%, and 5%, respectively, in different breeds of Tsaiya ducks. Altogether, a moderate to large genetic differentiation between populations suggests that the current studied duck populations have been maintaining a certain degree of within-population heterozygosity.

According to phylogenetic analysis, BWC and WHC were clearly separated from the Bangladeshi origin duck populations (BLD, RUP, and NAG), and JIN was grouped in a single cluster. This suggests a distinct genetic identity of these two populations, which is essential to establish as synthetic breeds. Interestingly, a close proximity between PEK and BWC, as well as WHC, can be explained by the introgression of PEK genetic material for higher productivity. Furthermore, these findings on WHC and BWC are supported by the considerable genetic distance seen in crossbred ducks originating from Pekin and Polish local duck populations [28]. Also, similar to the present findings, Pekin duck showed a distinct genetic profile from Tsaiya duck [13] and Chinese duck [37].

The structure analysis clarified the genetic architecture of the seven duck populations and supported the phylogenetic and pairwise genetic distance results. The most plausible clustering was at K = 4, where major genetic structures were revealed, with only minor improvement at K = 5. The findings of this paper are aligned with the reports of Lai et al. [13] in Tsaiya ducks and Wolc et al. [28] in Polish duck populations. PEK showed a distinct genetic structure compared with all other populations, which was similar to Lai et al. [13]. The similarity in genetic structure among JIN, NAG, BLD, and RUP supports the phylogenetic results and suggests recent gene flow among these populations. In contrast, BWC and WHC exhibited unique genetic structures compared to PEK and other indigenous populations, despite some introgression from PEK and NAG, which was also reflected in the heatmap, Nei’s distance, and pairwise analysis. The results demonstrated rich genetic diversity among the populations. The inbreeding coefficient (F_IS_) within populations at the marker level was 0.09, suggesting reduced extinction risk. A wide range of variability was observed within the populations.

## 5. Conclusions

This paper provides comprehensive information on the genetic background of seven duck populations of Bangladesh using microsatellite markers. Genetic diversity was found to be moderate (16%) among the studied populations, while the within-population heterozygosity accounted for the majority of variance (75%). The relatively low inbreeding coefficient (F_IS_ = 0.09) and wide allelic variability indicate a reduced risk of genetic erosion and considerable potential for future selection. The structure and phylogenetic analysis revealed the unique genetic identities of the BWC and WHC duck populations. As both crossbred populations were developed through approximately seven generations of controlled crossing, their gene and allele frequencies are expected to be relatively stable compared to early-generation crosses. The observed distinct genetic structure suggests that these populations can be considered as defined genetic groups and may serve as useful resources for future breeding and genetic improvement programs. These findings lay a solid foundation for the conservation of duck populations, the advancement of breed development, and the construction of innovative, sustainable breeding strategies that can retain the genetic diversity and improve the productivity of the genetic resources of Bangladesh.

## Figures and Tables

**Table 1:** Description of the microsatellite markers used in this paper.

Sl. No.	Marker Name	GenBank ID	Chr	Primer Sequence	Size	Dye
1.	CAUD111	AY587030	5	F: CTGACATTACACACCCAAACAGC	101	FAM
				R: CTTGACAAACTTTGGGAACAAGG		
2.	CAUD086	AY493331	1	F: CTGCATCCAAACTAGGCAGAC	187	VIC
				R: CAGACACCTACGGATATCGCTC		
3.	CAUD039	AY493284	1	F: CAGAGAGTGAAAGCTGCTGC	249	NED
				R: ACATGCCACTTGAACTTAATCAGGAA		
4.	CAUD069	AY493314	1	F: CTACTCAGTGTGCCTAATACCTC	349	PET
				R: GGTTAGTGAGTGAAGGGAGTT		
5.	APH04	AJ515884	6	F: GCTGCCTTCCACAACACTAC	132	VIC
				R: CGTCATGGTGCAGCTATCG		
6.	CAUD035	AY493280	6	F: GCTTCACTGCAGAACCAACTG	217	VIC
				R: CAAGCAGGTTGGCTTCCAG		
7.	APH20	AJ515895	8	F: CTGCGTTCATGACATTGTGAAGTG	277	VIC
				R: GAGGCTTTAGGAGAGATTGAAAAAGTAC		
8.	CAUD048	AY493293	11	F: CTGGACATCATTGCACAGACATAGT	336	VIC
				R: GACCTCAGAAATCTGCTGTTAGCT		
9.	APH08	AJ515887	6	F: CTGTGAAGCGAGCTAATTCAGC	107	FAM
				R: GTGTGCATCTGGGTGTGTATG		
10.	CAUD005	AY493250	1	F: CTGGCTGCTTCATTGCTGA	216	FAM
				R: CACATCTCAGGTCCTACAAGAC		
11.	CAUD040	AY493285	12	F: CTTGACGTTTCCTCACTGGAGA	350	NED
				R: GTTTACGCTGTGCGTGACTC		
12.	CAUD066	AY493311	1	F: GCAGGAAAGGAAGTGAGCC	179	FAM
				R: GCTCTGTTGCTCTTGTAACGAG		
13.	AMU3	AB180488	-	F: CTGAACCTGGCGGTATAAAGTGA	231	PET
				R: CTTCTTGGCAATGTCTTGAAGTGG		
14.	AMU68	AB180549	9	F: CACGAGGAACAGGACTACA	371	NED
				R: GAGCATACGATCCATGTCGG		

**Table 2:** Polymorphism information and F-statistics of the overall populations for each microsatellite marker used in this paper.

Locus ^1^	Allele Size (bp)	Na	He	Ho	F_IS_	F_IT_	F_ST_	HWE ^2^
CAUD111	91–103	5	0.52	0.55	−0.06	0.03	0.08	NS
AMU68	363–383	11	0.60	0.60	0.08	0.27	0.26	NS
APH04	119–137	7	0.68	0.69	−0.02	0.09	0.11	***
APH08	96–105	8	0.55	0.56	−0.02	0.29	0.32	NS
CAUD005	204–244	7	0.70	0.71	−0.02	0.04	0.05	NS
AMU3	230–236	4	0.53	0.48	0.09	0.28	0.21	*
CAUD069	307–355	15	0.62	0.18	0.07	0.08	0.27	***
CAUD086	174–188	6	0.65	0.69	−0.08	−0.01	0.06	NS
APH20	272–280	5	0.66	0.56	0.14	0.23	0.10	NS
CAUD066	172–184	7	0.64	0.52	0.20	0.35	0.20	NS
CAUD039	236–258	7	0.66	0.49	0.26	0.35	0.12	NS
CAUD040	316–404	26	0.88	0.85	0.04	0.10	0.06	NS
CAUD035	204–222	7	0.56	0.57	−0.07	0.22	0.23	NS
CAUD048	335–373	18	0.75	0.76	−0.01	0.10	0.10	NS
Mean		9.50	0.64	0.58	0.09	0.22	0.16	

^1^ Na, number of alleles; Ho, observed heterozygosity; He, expected heterozygosity; F_IS_, inbreeding within population; F_IT_, total inbreeding; F_ST_, genetic distance among populations; and HWE, Hardy–Weinberg equilibrium across studied duck populations of Bangladesh. ^2^ NS, Not significant; * and *** statistically significant deviation of HWE for *p* ≤ 0.05, and *p* ≤ 0.001, respectively.

**Table 3:** Genetic diversity measures of seven duck populations of Bangladesh using 14 microsatellite markers.

Population ^1^	N	Na	Ne	I	Ho	He	F
BLD	12	5.07 ± 0.82	3.46 ± 0.62	1.26 ± 0.14	0.53 ± 0.06	0.63 ± 0.04	0.15 ± 0.09
RUP	18	5.57 ± 0.21	3.74 ± 0.56	1.35 ± 0.13	0.61 ± 0.04	0.67 ± 0.04	0.08 ± 0.05
NAG	32	6.29 ± 1.08	4.14 ± 0.65	1.46 ± 0.12	0.59 ± 0.05	0.71 ± 0.03	0.15 ± 0.07
PEK	32	4.71 ± 0.77	2.49 ± 0.41	0.98 ± 0.13	0.51 ± 0.06	0.51 ± 0.05	0.01 ± 0.07
WHC	32	5.50 ± 0.95	3.46 ± 0.46	1.30 ± 0.12	0.59 ± 0.06	0.66 ± 0.03	0.12 ± 0.08
BWC	36	5.21 ± 0.67	3.10 ± 0.35	1.21 ± 0.11	0.63 ± 0.06	0.62 ± 0.04	0.03 ± 0.08
JIN	14	5.71 ± 0.75	3.85 ± 0.51	1.42 ± 0.10	0.64 ± 0.06	0.70 ± 0.02	0.10 ± 0.07
Mean	176	5.44 ± 0.31	3.46 ± 0.19	1.28 ± 0.05	0.59 ± 0.02	0.64 ± 0.02	0.09 ± 0.03

^1^ BLD, Non-descript Deshi; RUP, Rupali; NAG, Nageswari; PEK, Pekin; WHC, BAU White Crossbred; BWC, BAU Black and White Crossbred; and JIN, Jinding. N, number of observations; Na, average number of alleles; Ne, average number of effective alleles; I, Shannon’s information index; Ho, observed heterozygosity; He, expected heterozygosity; F, fixation index across loci for each population.

**Table 4:** Genetic distances (pairwise F_ST_) among seven different duck populations of Bangladesh.

	BLD	BWC	JIN	NAG	PEK	RUP	WHC
BLD	0						
BWC	0.208	0					
JIN	0.071	0.183	0				
NAG	0.046	0.167	0.039	0			
PEK	0.308	0.179	0.269	0.267	0		
RUP	0.054	0.186	0.073	0.043	0.271	0	
WHC	0.185	0.035	0.166	0.147	0.098	0.173	0

BLD, Non-descript Deshi; RUP, Rupali; NAG, Nageswari; PEK, Pekin; WHC, BAU White Cross; BWC, BAU Black and White Cross, and JIN, Jinding.

**Table 5:** Analysis of molecular variance based on the allelic distance matrix of F-statistics among seven duck populations of Bangladesh.

Source of Variation	d.f.	Sum of Squares	Mean Squares	Variance Components	F_ST_	Percentage of Variation	*p*-Value
Among populations	6	285.26	47.54	0.865	0.159	16	0.001
Among individuals	169	852.14	5.04	0.477		9	
Within individuals	176	719.50	4.08	4.088		75	
Total	351	1856.95		5.430		100	

## Data Availability

The original contributions presented in this paper are included in the article. Further inquiries can be directed to the corresponding authors.
